# Limited Associations between Keel Bone Damage and Bone Properties Measured with Computer Tomography, Three-Point Bending Test, and Analysis of Minerals in Swiss Laying Hens

**DOI:** 10.3389/fvets.2017.00128

**Published:** 2017-08-11

**Authors:** Sabine G. Gebhardt-Henrich, Andreas Pfulg, Ernst K. F. Fröhlich, Susanna Käppeli, Dominik Guggisberg, Annette Liesegang, Michael H. Stoffel

**Affiliations:** ^1^Federal Veterinary Office, Centre for Proper Housing of Poultry and Rabbits, Zollikofen, Switzerland; ^2^Agroscope, Institut für Lebensmittelwissenschaften (ILM), Bern, Switzerland; ^3^Vetsuisse Faculty, Institute of Animal Nutrition, University of Zürich, Zürich, Switzerland; ^4^Division of Veterinary Anatomy, Vetsuisse Faculty, University of Bern, Bern, Switzerland

**Keywords:** keel bone, laying hen, computer tomography, three-point bending, bone mineral content

## Abstract

Keel bone damage is a wide-spread welfare problem in laying hens. It is unclear so far whether bone quality relates to keel bone damage. The goal of the present study was to detect possible associations between keel bone damage and bone properties of intact and damaged keel bones and of tibias in end-of-lay hens raised in loose housing systems. Bones were palpated and examined by peripheral quantitative computer tomography (PQCT), a three-point bending test, and analyses of bone ash. Contrary to our expectations, PQCT revealed higher cortical and trabecular contents in fractured than in intact keel bones. This might be due to structural bone repair after fractures. Density measurements of cortical and trabecular tissues of keel bones did not differ between individuals with and without fractures. In the three-point bending test of the tibias, ultimate shear strength was significantly higher in birds with intact vs. fractured keel bones. Likewise, birds with intact or slightly deviated keel bones had higher mineral and calcium contents of the keel bone than birds with fractured keel bones. Calcium content in keel bones was correlated with calcium content in tibias. Although there were some associations between bone traits related to bone strength and keel bone damage, other factors such as stochastic events related to housing such as falls and collisions seem to be at least as important for the prevalence of keel bone damage.

## Introduction

Keel bone damage is a well-known welfare problem in laying hens (1–3). Käppeli et al. (4) established that 25.4% of Swiss laying hens had a moderately to strongly damaged keel bone (i.e., fractured) at the end of production. When weak deviations and small bumps were included, 55% of the animals were affected. These results are in agreement with other studies on this topic in various countries (5–10). Considering that a majority of moderate or severe keel bone deformities are healed fractures (11, 12) being associated with (chronic) pain (13, 14), this is a severe welfare issue.

Several causes may account for a keel bone deviation or fracture. Their relative contribution to keel bone damage is unknown. As one possibility, a fracture may result from an external trauma like, e.g., a crash or collision (15–19) or from strong compressive stress exerted on the keel bone by inappropriate perches (17, 20). In those cases, housing environments need to be optimized to avoid these events (9). Another possibility might be poor bone quality of hens as in osteoporosis due to inadequate dietary supplementation or excessive use of calcium for egg shell production. Osteoporosis may also affect other bones than keel bones and is defined as a decrease in completely mineralized structural bone which leads to increased fragility and probability of fracture (21). The link between osteoporosis and the prevalence of keel bone fractures in laying flocks is unclear.

The potential role of poor bone quality was investigated in this study by comparing damaged and healthy bones using peripheral quantitative computer tomography (PQCT) scans, and by examining the calcium and phosphorus content in the bone ash. Due to their irregular shape, keel bones cannot be examined by three-point bending tests. Assuming that calcium levels between keel bones and tibias are correlated, tibias were subjected to three-point bending tests. The sample was collected after slaughter and consisted of randomly picked laying hens from flocks varying in age, housing, and genetic origin. They reflected the pronounced variation in prevalence of keel bone damage found in Swiss flocks (4). It was tested whether the differences in bone properties correlated with the severity of keel bone damage. We hypothesized that intact keel bones would have a higher calcium content and greater cortical and trabecular densities than fractured keel bones. Furthermore, we predicted that the tibias of birds with intact keel bones would have a higher calcium content and greater shear strength and/or higher load–deformation curves than tibias of birds with fractured keel bones. The goal of the present study was to find out if future studies and efforts should focus on an improvement of bone quality and nutrition in addition to adjustments of the environment to prevent keel bone damage in laying hens. Since laying hens are of great economic importance and are kept in large groups of animals, the high prevalence means that a very large number of animals are affected. Therefore, studies about potential causes and methods to prevent keel bone damage are important.

## Materials and Methods

### Ethical Note

This research was done on laying hens after regular slaughter. No live animals were used in this study so no license for the ethical treatment was obtained.

### Bones

Left tibias and keel bones of 120 hens from 10 different flocks were collected from August through December 2009. These randomly sampled animals were a subsample of flocks that were used by Käppeli et al. (4). The hens had a mean age of 72 weeks ranging from 64 to 96 weeks. After the collection of the samples, the farm owners of the flocks were contacted to inquire about hybrid, color, age, housing system, outdoor access, material of perches, and flock size (Table 1). The data reflect the farmers’ statements. All samples were collected in the same commercial poultry abattoir, and the keel bones were palpated immediately after the defeathering process by the same examiner (Susanna Käppeli) for keel bone deviations and fractures. The repeatability of palpations was very high, and palpations were calibrated with another observer (4).

**Table 1:** Characteristics of the ten flocks that were used.

Flock	Housing	Perch	Hybrid	Range	Age	Mass	Fracture
1	Floor	Metal	LB	Yes	71	1,631	0.06
2	Aviary	Metal	HN	Yes	70	1,707	0.46
3	Aviary	Metal	HN	Yes	72	1,479	0.29
4	Aviary	Metal/plastic	LSL	Yes	70	1,527	0.29
5	Aviary	Metal/wood	LB	No	96	1,757	0.21
6	Aviary	Plastic	Silver	Yes	64	1,691	0.19
7	Aviary	Plastic	Silver	Yes	68	1,644	0.29
8	Aviary	Plastic	Silver	Yes	65	1,796	0.3
9	Aviary	Plastic	LSL	Yes	76	1,434	0.43
10	Aviary	Wood	LB	No	66	1,678	0.23

*HN, H&N Nick Chick (http://www.hn-int.com/eng/commercial-layers/nickchick.php); LB, Lohmann Brown (http://www.ltz.de/en/layers/alternative-housing/lohmann-brown-classic.php); LSL, Lohmann Selected Leghorn (http://www.ltz.de/en/layers/alternative-housing/lohmann-lsl-classic.php); Silver, Lohmann Silver (http://www.ltz.de/en/layers/alternative-housing/lohmann-silver.php); range = “yes” means that the hens had access to a veranda and a free range, the age is given in weeks, and the mean body mass in grams. Fracture (%) denotes the proportion of hens with fractured keel bones (palpation scores 1 and 2)*.

Palpation was performed by running two fingers down the edge of the keel bone to detect alterations like S-shaped deviations, bumps, depressions or proliferations. The following scoring system was used: 4 = intact keel bone, 3 = slight deviation, 2 = moderate deviation counted as fracture, 1 = severe deviation counted as fracture (4). For some analyses, scores 3 and 4 were combined as “no fractures” vs. scores 1 and 2 as “fractured.”

The defeathered bodies with feet, head, innards, and neck were weighed immediately after slaughter. The keel bone and the left leg including all the muscles were dissected from the rest of the hens’ bodies and frozen at −20°C. Sixteen weeks later, the samples were slightly thawed to remove all the muscles by using a scalpel and a hoof knife. Keel bones were then photographed in a standardized way lying on the right side. Thereafter, bones were immediately frozen again at −20°C.

Palpation with muscles attached may hide fractures. Therefore, all the photographs of keel bones were examined for fractures including the tip of the keel (by Sabine G. Gebhardt-Henrich), and the palpation score was corrected if needed. During initial palpation as well as during correction, the observers were unaware of the PQCT, three-point bending, and mineral ash content values of the bones.

### Peripheral Quantitative Computed Tomography

**Figure 1:**
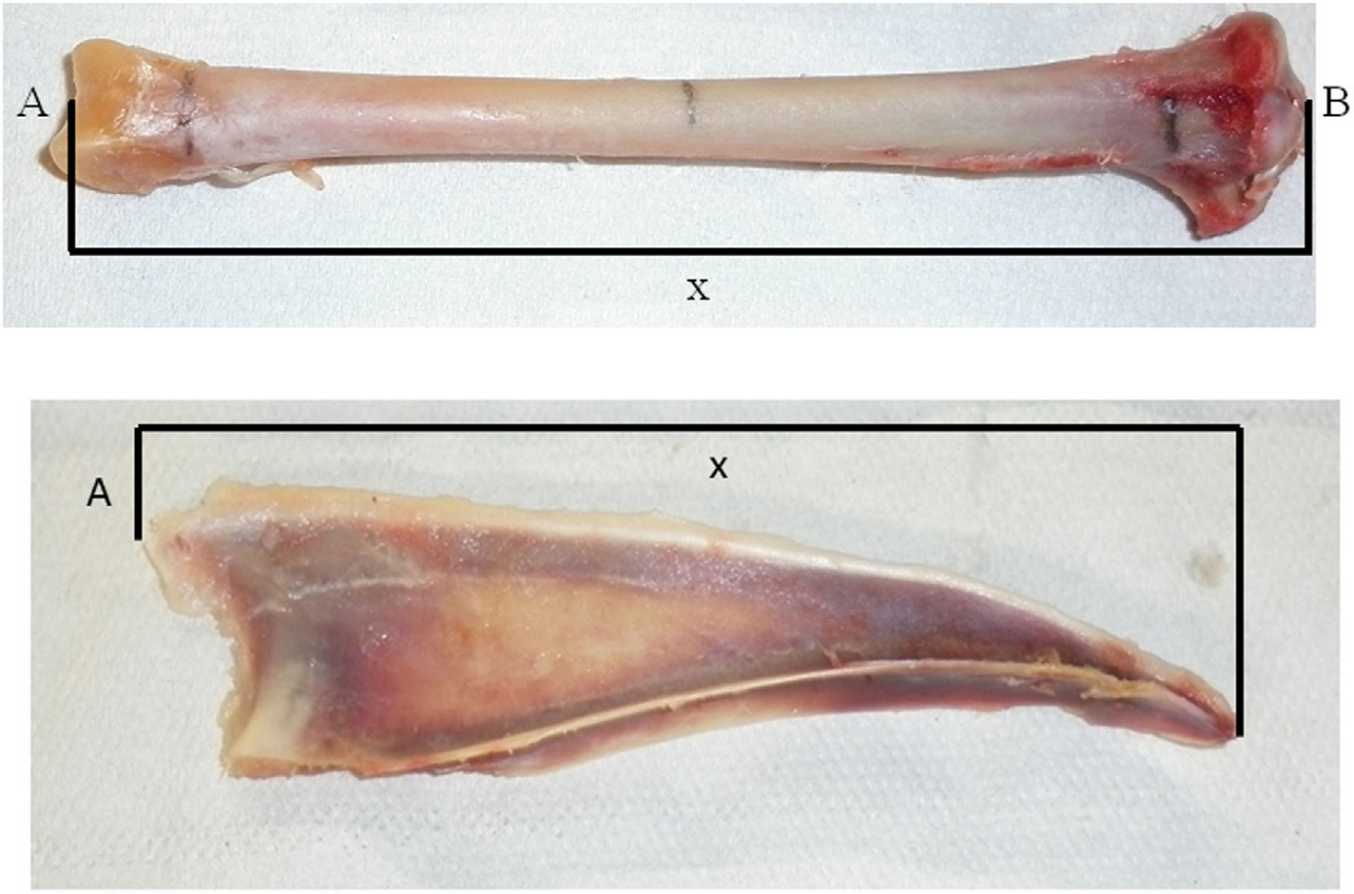
Points of measurement of the tibia (top) and the keel bone (bottom) for the peripheral quantitative computer tomography. Tibia: A: facies articularis inferior, B: eminentia intercondylaris, X: measured length of tibia. Keel bone: A: apex carinae, X: length of carina sterni.

After the keel bones and the tibias had been thawed to room temperature, they underwent scans by PQCT with a Stratec™ XCT 960A (Stratec Medizintechnik GmbH, Pforzheim, Germany). The PQCT was tested for accuracy and validity in several species, and the results were replicable (22). This technique gives further insight into the bone structure and mineral content. Proximal, distal, and central measuring points at 10, 50, and 90% of the distance between the proximal and distal extremities of the tibiotarsus and the overall length of the carina sterni of the keel bones were determined with digital calipers (Figure 1). The total, cortical, and trabecular mineral contents and densities were calculated by the computer program as based on the attenuation of the X-rays (23). Afterward, bones were frozen again at −20°C. The center and the distal position of keel bones involving two birds of one flock and the distal position of a keel bone of a bird from another flock could not be measured because the bones were too weak and the computer could not calculate the value since it was below the detection limit.

### Three-Point Bending Test

After thawing the tibias for at least 24 h at 15°C, they were broken with the *Three-Point Bending Test of Animal Bone* following the ASABE Standards 2007 [ANSI/ASAE S459 MAR1992 (R2007)] using a Zwick and Roell universal testing machine with a 2.5 kN load cell. The fulcrum was adjusted at 55 mm to get the requested length to bone diameter ratio greater than 10. We measured the smallest diameter of the tibias. The bones were laid in the test apparatus with the flattest side down, and the force was applied to the midshaft with a crosshead speed of the loading bar of 10 mm/min. The force–deformation curve was read from the texture analyzer (24). From this curve, the ultimate force *F* required to break a bone was recorded in Newton. To take bone size into account, the value of the ultimate force was divided by the cross section of the bone (*A*), which was calculated as the product of π and the square of the radius of the thinnest part of the bone (using the small diameter, see ANSI/ASAE 8.2). This (*F*/2 × *A*) was taken as an approximation of the ultimate shear strength. Bones were too thin to measure the outer and inner diameter to get a more exact measurement of ultimate shear strength. Bending strength (force applied when the bone fractures) was automatically derived from the slope of load/displacement graph (25, 26) and the slope of the load–deformation curve, which is an estimate for the bone stiffness, was derived by the regression between 0.3 and 0.5 mm.

### Bone Ash

The tibias and keel bones were ashed in porcelain crucibles in a muffle furnace (N11/R, Fabr. Nr. 70766, “Tony Güller” NABER Industrieofen, Lilienthal, Germany) for 24 h at 550°C. Before and after ashing, the bones were weighed, and the remaining mass was taken as the bone mineral ash content. The contents of phosphorus and calcium in the ash were further analyzed with a Cobas Mira Plus (Roche Diagnostic, Basel, Switzerland) to yield the calcium and phosphorus contents.

### Statistics

The residuals of the analyses were visually inspected for the assumption of normality. Full models including color of the hybrid (white, brown, and silver), age, and body mass were analyzed with general linear models (MIXED Procedure of SAS^®^) with flock as the subject and the 12 hens per flock as repeated measures. Flock nested in color of hybrid was taken as a random variable. Since the genetics, behavior, and production traits are similar for the hybrids LSL and HN, they were pooled as white hybrids. Initially, all interactions were included in the models. For the final model, interactions >0.2 were removed. *Post hoc* tests were carried out with a Bonferroni adjustment. Correlations with CT measurements are Pearson or Spearman rank correlations as indicated by the subscripts.

Some distal PQCT measurements of keel bones were missing because fractures were located at these positions of the corresponding bones. Therefore, the measurements at the center were used in the subsequent analyses. The PQCT measurements were logarithmically transformed to fulfill the assumption for parametric analyses.

The ultimate shear strength and the slope of the load–deformation curve were logarithmically transformed for general linear model analyses but not for the Spearman rank correlations. Since ultimate shear strength was adjusted for the size of the bone and therefore bird size, mass was not included as a factor analyzing this parameter. The slope of the load–deformation curve, however, was not adjusted to bone size and, therefore, the body mass of the bird was taken as a factor into the model.

There was only one flock kept on floor housing instead of in aviaries. Analyses were performed with and excluding this flock. Results are given for the complete data set, but differences in significances are noted when this flock was excluded.

## Results

**Figure 2:**
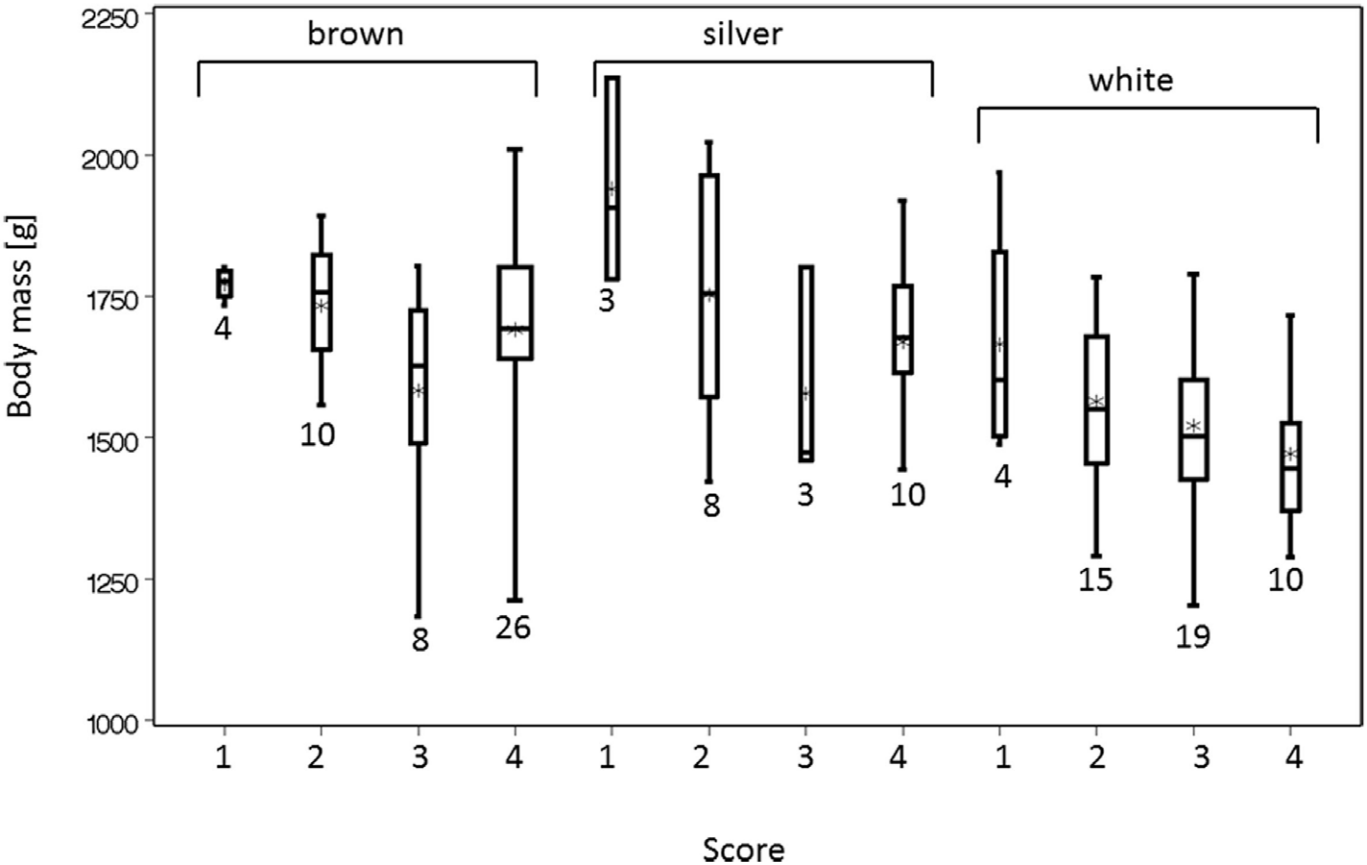
Body mass after slaughter was associated with the score of the keel bone. Score 4 indicates an intact keel bone, score 3 indicates a slightly deformed keel bone, and scores 2 and 1 (=worst) indicate fractured keel bones. The width of the box plots indicates the sample size, and the numbers of animals are beneath the bars.

In this sample of 120 keel bones, 36 (30%) were scored as intact (score 4), 37 (31%) were slightly deformed (score 3), 35 (29%) were moderately (score 2), and 12 (10%) were severely fractured (score 1). In 9 out of 76 cases, keel bone fractures were detected on the photographs that were not detected by palpation. In four cases, a fracture was suspected during palpation but was not seen on the photographs. Body mass after bleeding in the abattoir was associated with keel bone scores. Hens with more severely damaged keel bones were heavier than hens with more slightly deviated keel bones (i.e., deformations without obvious callous material) whereas hens with intact keel bones where of intermediate mass in brown and silver hens but lowest in white hens (Figure 2). In the analysis of keel bones excluding score 4 (intact keel bone), body mass was significantly different between hens with different scores, and there was an influence of both color of hybrid and age with an interaction between age and color of hybrid (keel bone score: *F*_1,63_ = 11.83, *P* < 0.001; color of hybrid: *F*_2,4_ = 9.87, *P* = 0.028; age: *F*_1,4_ = 12.71, *P* = 0.024; color of hybrid × age: *F*_2,4_ = 10.45, *P* = 0.026, *N* = 74 hens in 10 flocks). Body mass of hens with keel bone scores of 1 and 2 differed from the body mass of hens with keel bone score 3 (*F*_1,63_ = 11.83, *P* < 0.001). In the model including the intact keel bones, both keel bone score (*F*_1,109_ = 5.88, *P* < 0.015) and age (*F*_1,4_ = 8.99, *P* < 0.04) were associated with variation in body mass. There was a trend for color of hybrid (*F*_2,4_ = 6.85, *P* = 0.051) and an interaction between age and color of hybrid (*F*_2,4_ = 7.64, *P* = 0.043), but the fit of the model was worse: the corrected Akaike’s information criterion was 1,497 instead of 889 in the model without intact keel bones. A higher Akaike’s information criterion means that the model fit is poor (27) (Figure 2).

Body mass was not correlated with age (*r*_P_ = −0.07, NS, *N* = 10 flocks).

### Computer Tomography

The total bone mineral contents of the keel bone and the tibia were positively correlated (*r*_P_ = 0.25, *P* = 0.006, *N* = 119). The center measurements of all measured bone traits were positively correlated with the distal and proximal measurements, and the mineral contents and densities of trabecular and cortical bone were positively correlated with body mass in most cases (Table 2). The values of the PQCT measurements of the keel bones and the tibias in relation to the palpation scores are shown in Table 3. Cortical and trabecular bone mineral contents were lower in intact keel bones (score 4) than in damaged keel bones (cortical: *F*_1,8_ = 8.55, *P* = 0.02; trabecular: *F*_1,8_ = 8.25, *P* = 0.02). Trabecular content but not cortical content differed among colors of hybrids (*F*_2,6_ = 5.46, *P* = 0.05). Without the farm with floor housing, cortical content but not trabecular content was lower in white than in brown and silver hybrids (cortical content *F*_2,5_ = 5.8, *P* = 0.05; trabecular content *F*_2,5_ = 4.75, *P* = 0.07). Age and body mass were not associated with cortical or trabecular content. Keel bones with different scores did not differ in density measurements, but white hybrids had lower densities than brown and silver hybrids and age was positively associated with the density measurements (cortical density: color of hybrid *F*_2,6_ = 18.21, *P* = 0.003; age *F*_1,6_ = 25.17, *P* = 0.002, without the farm with floor housing there is an interaction between mass and presence of fracture *F*_1,94_ = 4.46, *P* = 0.04; trabecular density: color of hybrid *F*_2,6_ = 5.5, *P* = 0.04, without the farm with floor housing *F*_2,5_ = 4.81, *P* = 0.07; age *F*_1,6_ = 6.62, *P* = 0.04). Body mass was not associated with PQCT density measurements (Tables 2 and 3).

**Table 2:** Spearman’s correlations between the different PQCT measurements and body masses.

Trait	Center with proximal KB	Center with proximal TB	Center with distal KB	Center with distal TB	Body mass with KB	Body mass with TB
Total bone MC	0.39***	0.66***	0.21*	0.59***	0.36***	0.44***
Total bone MD	0.73***	0.71***	0.74***	0.57***	0.20*	−0.09
Trabecular bone MC	0.38***	0.56***	0.32**	0.29***	0.35***	0.0
Trabecular bone MD	0.62***	0.65***	0.58***	0.40***	0.17^(*)^	−0.18*
Cortical bone MC	0.43***	0.71***	0.38***	0.60***	0.32**	0.37***
Cortical bone MD	0.71***	0.71***	0.63***	0.66***	0.19*	0.02

^(*)^<0.1, *<0.5, **<0.01, ***<0.001, *N* = 116–120.

*KB, keel bone; TB, tibia; MC, mineral content; MD, mineral density*.

*Proximal, distal, and central measuring points refer to 10, 50, and 90% of bone length*.

**Table 3:** Measurements of the total bone mineral content (mg/cm), total bone mineral density (mg/cm^3^), trabecular bone mineral content (mg/cm), trabecular bone mineral density (mg/cm^3^), cortical bone mineral content (mg/cm), and cortical bone mineral density (mg/cm^3^) by peripheral quantitative computer tomography (see Materials and Methods).

	Color	P = 4	P = 3	P = 2	P = 1	SE
**(A) Keel bone**						

Total bone mineral content	White	9.1	14.1	16.8	25.3	3.32
	Brown	14.4	21.6	25.6	23.0	
	Silver	14.3	17.6	22.8	26.2	

Total bone mineral density	White	385.7	409.0	458.9	497.2	31.58
	Brown	413.2	352.8	449.0	333.5	
	Silver	457.5	513.9	499.7	536.5	

Trabecular bone mineral content	White	4.1	6.2	8.8	13.1	1.73
	Brown	6.8	11.6	13.3	11.2	
	Silver	6.9	8.9	11.3	13.1	

Trabecular bone mineral density	White	416.7	432.1	535.3	579.1	37.74
	Brown	453.2	426.3	532.8	371.0	
	Silver	503.4	581.0	558.2	596.6	

Cortical bone mineral content	White	7.4	12.8	15.2	23.1	3.30
	Brown	12.6	20.8	22.5	20.9	
	Silver	12.4	15.9	20.7	23.7	

Cortical bone mineral density	White	447.23	488.7	503.0	532.5	22.73
	Brown	472.636	433.6	507.9	412.9	
	Silver	494.07	558.5	532.9	586.9	

**(B) Tibia**						

Total bone mineral content	White	28.6	28.7	28.3	28.0	0.48
	Brown	34.5	31.9	34.6	34.7	
	Silver	31.0	31.2	33.5	33.5	

Total bone mineral density	White	510.0	498.4	482.1	477.4	6.33
	Brown	442.3	419.4	466.9	429.6	
	Silver	427.5	463.0	444.0	461.8	

Trabecular bone mineral content	White	8.2	8.3	8.0	6.8	0.20
	Brown	9.0	8.0	9.1	9.3	
	Silver	6.6	7.5	8.0	8.4	

Trabecular bone mineral density	White	334.9	328.8	308.1	267.3	6.70
	Brown	253.1	232.3	270.7	253.9	
	Silver	203.1	245.2	234.0	260.9	

Cortical bone mineral content	White	25.9	26.1	25.6	24.1	0.46
	Brown	30.9	29.0	30.9	32.5	
	Silver	26.8	27.0	28.8	28.4	

Cortical bone mineral density	White	651.2	640.7	624.3	660.0	6.26
	Brown	596.2	566.9	629.2	539.2	
	Silver	632.9	652.5	628.4	635.0	

*Only the measurement of the center of the keel bone was used. Mass denotes body mass. For the tibia, the means of the three measurements of this bone are shown in the table. Non-significant interactions are not shown*.

*Color, color of hybrid; P, palpation score*.

There was clearly no association between the PQCT measurements of the tibia and the fracture status of the keel bone except for trabecular content, which increased with degree of keel bone damage (*F*_1,8_ = 8.25, *P* = 0.02). Body mass, however, had a positive association with cortical content (*F*_1,108_ = 12.79, *P* = 0.0005) and with trabecular density (*F*_1,108_ = 14.47, *P* = 0.0002) and brown hybrids had lower cortical density (*F*_2,6_ = 7.26, *P* = 0.025) but higher trabecular content (*F*_2,6_ = 5.46, *P* = 0.045) than white and silver hybrids. When excluding the farm with floor housing, the color of hybrid did not differ for trabecular content (*F*_2,5_ = 4.75, *P* = 0.07).

### Three-Point Bending Test

Color of hybrid and age but not keel bone score explained variation in ultimate shear strength required to break the tibia (keel bone score: *F*_1,109_ = 1.47, NS; color of hybrid: *F*_2,6_ = 56.61, *P* < 0.0001; age: *F*_1,6_ = 16.44, *P* = 0.007). White hens had a higher ultimate shear strength (3.73 kN) than LB (2.84 kN) or silver hybrids (2.83 kN) (SE = 0.08, *F*_1,6_ = 99.81, *P* < 0.0001). However, ultimate shear strength was significantly higher in birds with intact than in fractured keel bones (least squares means intact: 3.53 N/mm^2^ ± 0.11, fractured: 3.10 ± 0.05, *F*_1,8_ = 14.25, *P* < 0.0001). Likewise, the individual keel bone score did not explain variation in the load–deformation curve. The load–deformation curve did not differ in hens with or without fractured keel bones but was positively associated with body mass and white hybrids had lower values (184.66 kN/mm^2^) than brown (208.73 kN/mm^2^) and silver hybrids (217.65 kN/mm^2^) (keel bone score: *F*_1,106_ = 0.15, NS; color of hybrid: SE = 4.58, *F*_2,6_ = 6.99, *P* = 0.03; age: *F*_1,6_ = 5.33, *P* = 0.06; body mass: *F*_1,106_ = 14.81, *P* = 0.0002). Disregarding the one farm with floor housing, the load–deformation curve increased with age (*F*_1,5_ = 6.72, *P* = 0.05).

### Bone Mineral Ash

**Figure 3:**
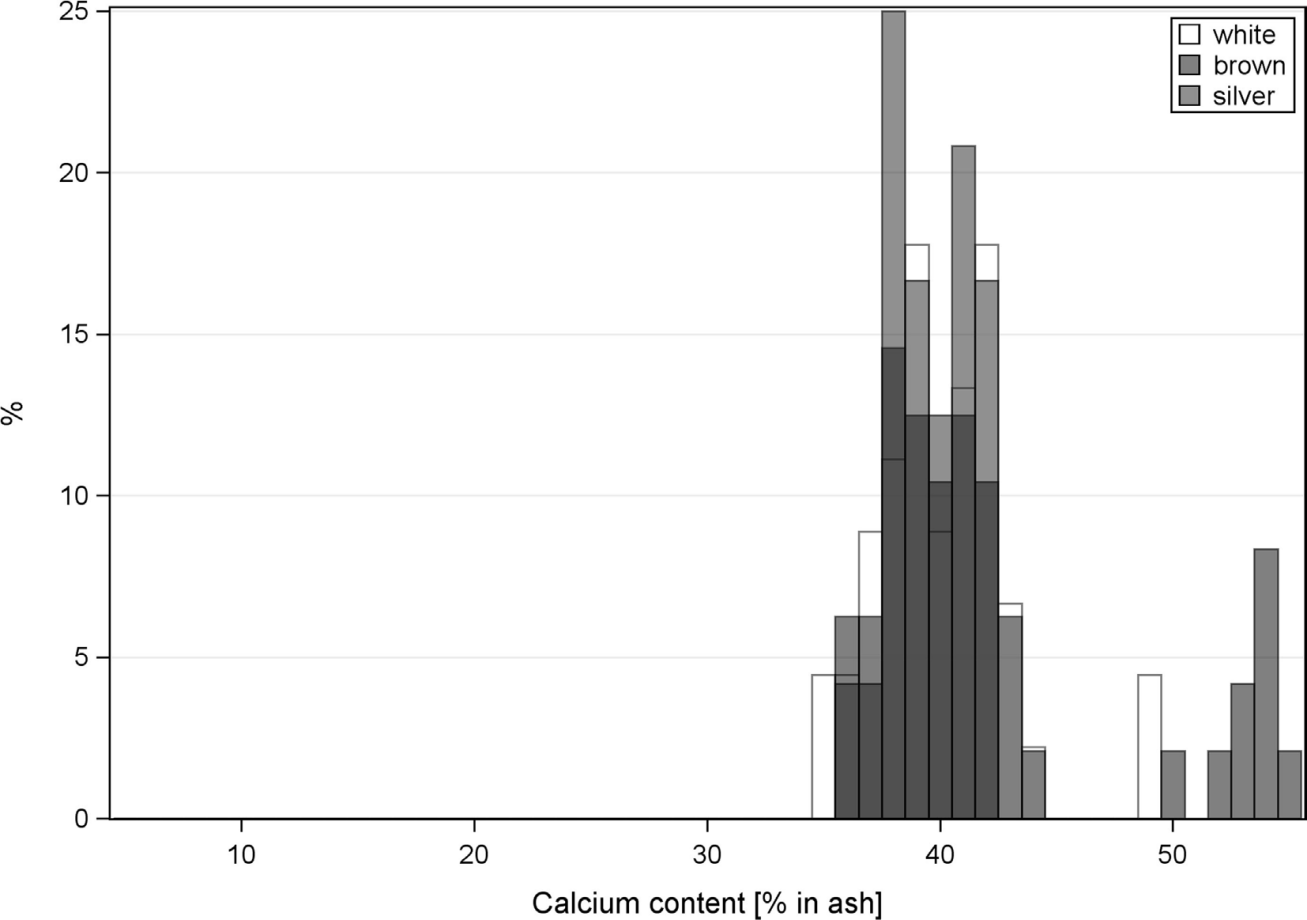
Histogram of the calcium content of keel bones (a) and tibia (b). White hens have white bars, brown hens have black bars, and silver hens have gray bars.

**Figure 4:**
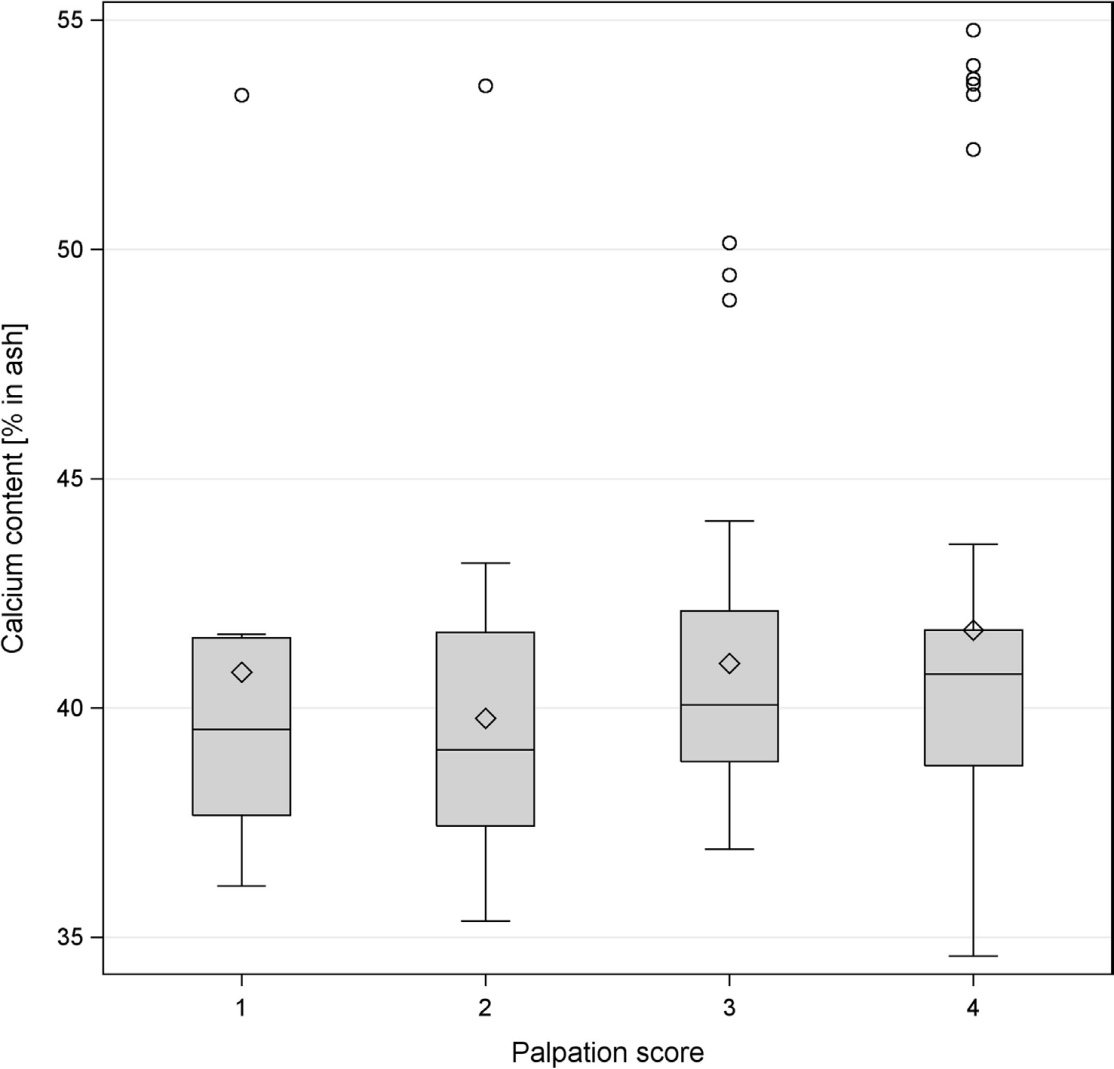
Boxplot of the levels of percentage of calcium content in the keel bone and palpation score of the same bone. The horizontal line shows the median, and the diamonds show the mean. The levels of calcium content were classified as follows: level 1 <38%, level 2 38–40.36%, level 3 40.37–42%, level 4 >42%.

The calcium content in keel bones and to a lower degree in tibias had a distinct bimodal distribution (Figure 3). Intact keel bones (palpation score 4) and keel bones with slight deviations (palpation score 3) had significantly higher calcium content and significantly higher mineral content in the keel bone than keel bones with moderate and severe damage (palpation scores 1 and 2)(calcium: 14.96% in ash ±0.69 vs. 13.90 ± 0.70, *F*_1,102_ = 4.16, *P* < 0.04; color: *F*_2,7_ = 0.25, NS, Figure 4). The calcium content in the keel bone and the tibia were positively correlated (tibia: *F*_1,105_ = 26.35, *P* < 0.0001; color: *F*_2,6_ = 0.04, NS; age: *F*_1,105_ = 0.26, NS). The proportion of calcium to phosphorus in the tibia was negatively associated with the ultimate force needed to bend the tibia (estimate of Ca/P: −0.59 ± 0.22, *F*_1,102_ = 7.4, *P* = 0.008; body mass: *F*_1,102_ = 4.76, *P* = 0.03; age: *F*_1,8_ = 0.07, NS; Ca/P × age: *F*_1,102_ = 6.53, *P* = 0.01; body mass × age: *F*_1,102_ = 5.55, *P* = 0.02) (Figures 3 and 4). Body mass was not associated with calcium content in keel bones (*F*_1,104_ = 0.13, NS).

## Discussion

Considering the tremendous number of affected animals with keel bone damage (4) and the evidence that these animals suffer from pain (13, 14), it is crucial to address the causes and to seek solutions to this problem. It is self-evident to assume that the detected fractures occurred in the live animals. In the larger data set, some flocks were palpated on the farm at depopulation and there were no differences in the prevalence of fractures between flocks at the farm or the abattoir (4). Furthermore, the prevalence in our sample is in agreement with other studies on live hens in the barn before depopulation (9, 16–18). Callous material at the site of fractures also support the time of fracture in live birds. The variation in housing regarding the material of perches, the presence or absence of a free range, different aviary designs or floor housings as well as a range of hybrids and different ages at slaughter reflects the population of Swiss laying hens at the abattoir. In this sample, intact keel bones had a higher calcium content, and the tibias of those birds had a greater shear strength than keel bones and tibias from birds with fractured keel bones. Although relationships without experimental intervention cannot elucidate causation, associations may help to identify predictive indicators for bone damage that can be investigated in future studies.

Contrary to Donaldson et al. (28) who did not find an association between body mass and keel bone score, our sample revealed that hens with more severely affected keel bones were heavier than hens with more slightly deformed keel bones and hens with intact keel bones were of intermediate mass (Figure 2). This could be one indication for a traumatic origin of keel bone injuries by falls and collisions as other studies suggest (16–19). The incidence of having an accident may be independent of body mass but when a fall or collision occurs, the probability of suffering a deviation or fracture of a certain severity is likely to be proportional to body mass. Hens with intact keel bones that do not have accidents have an average body mass. This is to be expected if the likelihood of falling and crashing is independent of body mass. We explain the higher severity of fractures in heavier hens with the fact that the impact of a trauma is bigger with increasing collision energy. This is consistent with the model used by Toscano et al. (29). In the study of Donaldson et al. (28), falls and collisions did not seem to contribute significantly to keel bone injuries as the presence of perches did not influence the prevalence of keel bone damage [but see Ref. (16)]. Notwithstanding, material and design of perches seem to be important causative factors for keel bone damage as was shown by Käppeli et al. (30) and Stratmann et al. (18). Like in falls, more force would act on the keel bone during perching in heavier birds compared with lighter birds.

Alternatively, larger hens might lay larger eggs and a different calcium metabolism might make osteoporosis more likely. In our data set, mass was clearly not associated with calcium content in keel bones.

Unexpectedly, the PQCT measurements of keel bones showed significantly lower cortical and trabecular bone mineral contents in intact compared with deformed keel bones. This might be explained by the fact that a bone fracture initiates a healing process. It goes along with the formation of a fracture callus in which a higher mineral content can be found because of bone remodeling of the callus during the healing process (31). However, the calcium content of the entire bone as determined by ashing showed the opposite pattern in that intact keel bones had a higher overall calcium content. Thus, the PQCT data of the already fractured keel bones did not give us indications of keel bone strength *before* the incidence of deviation/fraction, although the site of measurement was not directly affected by visible fractures. This is in contrast to the study by Tarlton et al. (32) where keel bones with greater bone density and bone mineral content had a lower breaking rate and less severe keel bone damages. Using experimentally induced fractures of keel bones, Toscano et al. (29) found a negative relationship between keel surface bone mineral density and the severity of fracture. Assuming a correlation between bone strength of keel bones and tibias (25), we wanted to avoid this problem by examining the tibias by PQCT hoping that this would give us some information on the keel bone parameters before fracture. However, the PQCT measurements of the tibia did not correlate with the palpation scores of the keel bones (Table 3B). Therefore, PQCT measurements of tibias seem to be an inappropriate predictor for keel bone deviations or fractures but are useful for the assessment of bone mineral deviations in the skeletal system.

In the three-point bending test, the ultimate shear strength and the load–deformation curve were determined. The ultimate shear strength indicates the maximum load a bone can sustain (33). A high load–deformation curve indicates that a bone is more rigid and less ductile, whereas a low load–deformation curve implies that the bone is more ductile and less mineralized (34, 35). Bone mineral density was found to correlate positively between keel bones and tibias (29). Although birds with intact keel bones (score 4) had significantly higher ultimate shear strengths than those with fractured keel bones, neither the ultimate shear strength nor the load–deformation curve could be used as an indicator for the individual palpation score in our examination. This partly agrees with the studies by Fleming et al. (8) and Donaldson et al. (28) who found a correlation between keel bone score and tibia bone strength. In their study, however, no *post hoc* contrasts were calculated. Thus, it remains unclear whether their significance was mainly due to the difference between damaged and undamaged keel bones or due to damages of different severity. After supplementing laying hens with n3, keels had a higher load at failure, and tibia and humeri were more flexible (36). The fact that the load–deformation curve and ultimate shear strength were not associated with the severity of keel bone damage in our study again suggests a possible traumatic origin from the environment for keel bone fractures and supports the notion of falls and collisions as causes (16–18). Beyond the effect of having an accident that was presumably independent of bone condition, bone strength seemed to have some influence on the probability of fractures as revealed by an increased ultimate shear strength in birds with intact keel bones. The different causes of fractures would make correlations between bone strength and bone damage weak and spurious.

Bone mineral ash content analyses were consistent with our expectations of bones from birds with less damaged keel bones having higher mineral content and calcium content, respectively, as reported in the studies by Fleming et al. (8) and Donaldson et al. (28). The importance of calcium is also supported by the fact that the six highest calcium values of keel bones are from the farm with the lowest prevalence of keel bone fractures (flock 1, Table 1). The calcium/phosphorus ratio was negatively associated with bone strength confirming that both minerals are essential for bone strength (37).

In agreement with Regmi et al. (2), hybrids differed in bone properties. All the investigated birds were kept cage-free so no influences of housing conditions on bone properties were expected, which were found between cage-kept and cage-free kept laying hens (38, 39).

As suggested by Casey-Trott et al. (31), the accuracy of live palpations is limited, and in 12% of the keel bones, fractures were missed that were detected on dissected keel bones, and in 9%, palpated fractures could not be corroborated on the photographs.

The main conclusion of our study is that bone properties such as density, bending force, ash and mineral contents explain only a limited proportion of the variance of the prevalence and severity of keel bone damage. Thus, environmental factors such as housing, perch design, and accidents may present the most likely causes for fractures. An alternative explanation for the lack of correlations between bone properties measured by PQCT and keel bone condition could be that our bone measurements at depopulation did not represent the bone conditions at the time of fracture. Keel bone deviations and fractures might have changed the bone properties to such an extent that PQCT did not yield usable results. Using old hens that were well past the peak laying rate might not have given us the bone properties at the age at peak laying when fractures occurred (29, 40). However, the high values in calcium content in certain flocks along with the low number of fractures in these flocks indicate that mineralization and structure of the bones may affect the prevalence of keel bone fractures. Therefore, further research in this direction is needed.

## Author Contributions

SG-H—conceived the study, analyzed the data, and wrote the manuscript; AP—collected the data and wrote the manuscript; EF—conceived the study; SK—collected the data; DG—conceived the study, planned and helped with the three-point bone breaking test; AL—conceived the study, planned and helped with the PQCT; MS—conceived the study and supervised the DVM thesis of AP.

## Conflict of Interest Statement

The authors declare that the research was conducted in the absence of any commercial or financial relationships that could be construed as a potential conflict of interest. The reviewer, TR, and handling editor declared their shared affiliation, and the handling editor states that the process nevertheless met the standards of a fair and objective review.

## References

[1] Harlander-Matauschek A Rodenburg TB Sandilands V Tobalske BW Toscano MJ Causes of keel bone damage and their solutions in laying hens. Worlds Poult Sci J (2015) 71(03):461–72. 10.1017/S0043933915002135

[2] Regmi P Nelson N Steibel JP Anderson KE Karcher DM Comparison of bone properties and keel deformities between strains and housing systems in end-of-lay hens. Poult Sci (2016) 95(10):2225–34. 10.3382/ps/pew199 27433008

[3] Sandilands V Moinard C Sparks NHC. Providing laying hens with perches: fulfilling behavioural needs but causing injury? Br Poult Sci (2009) 50(4):395–406. 10.1080/00071660903110844 19735008

[4] Käppeli S Gebhardt-Henrich SG Fröhlich E Pfulg A Stoffel MH Prevalence of keel bone deformities in Swiss laying hens. Br Poult Sci (2011) 52(5):531–6. 10.1080/00071668.2011.615059 22029778

[5] Bestman M Wagenaar J Farm level factors associated with feather pecking in organic laying hens. Livest Prod Sci (2003) 80:133–40. 10.1016/S0301-6226(02)00314-7

[6] Budgell KL Silversides FG Bone breakage in three strains of end-of-lay hens. Can J Anim Sci (2004) 84(4):745–7. 10.4141/a04-040

[7] Elson HA Croxall R European study on the comparative welfare of laying hens in cage and non-cage systems. Arch Geflügelk (2006) 70(5):194–8.

[8] Fleming RH McCormack HA McTeir L Whitehead CC. Incidence, pathology and prevention of keel bone deformities in the laying hen. Br Poult Sci (2004) 45(3):320–30. 10.1080/00071660410001730815 15327118

[9] Heerkens J Delezie E Rodenburg TB Kempen I Zoons J Ampe B Risk factors associated with keel bone and foot pad disorders in laying hens housed in aviary systems. Poult Sci (2016) 95(3):482–8. 10.3382/ps/pev339 26628344

[10] Silversides FG Singh R Cheng KM Korver DR. Comparison of bones of 4 strains of laying hens kept in conventional cages and floor pens. Poult Sci (2012) 91(1):1–7. 10.3382/ps.2011-01453 22184423

[11] Scholz B Rönchen S Hamann H Distl O Bone strength and keel bone status of two layer strains kept in small group housing systems with different perch configurations and group sizes. Berl Munch Tierarztl Wochenschr (2009) 122(7/8):249–56. 19681397

[12] Wilkins LJ Brown SN Zimmerman PH Leeb C Nicol CJ. Investigation of palpation as a method for determining the prevalence of keel and furculum damage in laying hens. Vet Rec (2004) 155(18):547–9. 10.1136/vr.155.18.547 15559420

[13] Nasr M Nicol C Murrell J. Do laying hens with keel bone fractures experience pain? PLoS One (2012) 7(8):e42420. 10.1371/journal.pone.0042420 22927930 PMC3425496

[14] Nasr M Browne WJ Caplen G Hothersall B Murrell JC Nicol CJ Positive affective state induced by opioid analgesia in laying hens with bone fractures. Appl Anim Behav Sci (2013) 147(1–2):127–31. 10.1016/j.applanim.2013.04.015

[15] Lay DC Jr Fulton RM Hester PY Karcher DM Kjaer JB Mench JA Hen welfare in different housing systems. Poult Sci (2011) 90(1):278–94. 10.3382/ps.2010-00962 21177469

[16] Wilkins LJ McKinstry JL Avery NC Knowles TG Brown SN Tarlton J Influence of housing system and design on bone strength and keel bone fractures in laying hens. Vet Rec (2011) 169(16):414. 10.1136/vr.d4831 21862469

[17] Stratmann A Fröhlich EK Harlander-Matauschek A Schrader L Toscano MJ Würbel H Soft perches in an aviary system reduce incidence of keel bone damage in laying hens. PLoS One (2015) 10(3):e0122568 10.1371/journal.pone.0122568 25811980 PMC4374857

[18] Stratmann A Fröhlich EK Gebhardt-Henrich SG Harlander-Matauschek A Würbel H Toscano MJ Modification of aviary design reduces incidence of falls, collisions and keel bone damage in laying hens. Appl Anim Behav Sci (2015) 165:112–23. 10.1016/j.applanim.2015.01.012

[19] Campbell D Goodwin SL Makagon MM Swanson JC Siegford JM. Failed landings after laying hen flight in a commercial aviary over two flock cycles. Poult Sci (2016) 95(1):188–97. 10.1093/ps/pev270 26527703

[20] Pickel T Schrader L Scholz B. Pressure load on keel bone and foot pads in perching laying hens in relation to perch design. Poult Sci (2011) 90(4):715–24. 10.3382/ps.2010-01025 21406354

[21] Whitehead CC Fleming RH. Osteoporosis in cage layers. Poult Sci (2000) 79(7):1033–41. 10.1093/ps/79.7.1033 10901207

[22] Gasser JA. Assessing bone quantity by pQCT. Bone (1995) 17(4):S145–54. 10.1016/8756-3282(95)00287-N 8579910

[23] Islam KMS Schaeublin H Wenk C Wanner M Liesegang A. Effect of dietary citric acid on the performance and mineral metabolism of broiler. J Anim Physiol Anim Nutr (Berl) (2012) 96(5):808–17. 10.1111/j.1439-0396.2011.01225.x 22093035

[24] Baird HT Eggett DL Fullmer S. Varying ratios of omega-6: omega-3 fatty acids on the pre-and postmortem bone mineral density, bone ash, and bone breaking strength of laying chickens. Poult Sci (2008) 87(2):323–8. 10.3382/ps.2007-00186 18212376

[25] Bishop SC Fleming RH McCormack HA Flock DK Whitehead CC. Inheritance of bone characteristics affecting osteoporosis in laying hens. Br Poult Sci (2000) 41(1):33–40. 10.1080/00071660086376 10821520

[26] Fleming RH McCormack HA McTeir L Whitehead CC. Relationships between genetic, environmental and nutritional factors influencing osteoporosis in laying hens. Br Poult Sci (2006) 47(6):742–55. 10.1080/00071660601077949 17190683

[27] Bozdogan H Model selection and Akaike’s Information Criterion (AIC): the general theory and its analytical extensions. Psychometrika (1987) 52(3):345–70. 10.1007/BF02294361

[28] Donaldson CJ Ball MEE O’Connell NE. Aerial perches and free-range laying hens: the effect of access to aerial perches and of individual bird parameters on keel bone injuries in commercial free-range laying hens. Poult Sci (2012) 91(2):304–15. 10.3382/ps.2011-01774 22252341

[29] Toscano MJ Wilkins LJ Millburn G Thorpe K Tarlton JF. Development of an ex vivo protocol to model bone fracture in laying hens resulting from collisions. PLoS One (2013) 8(6):e66215. 10.1371/journal.pone.0066215 23785487 PMC3681979

[30] Käppeli S Gebhardt-Henrich SG Fröhlich EK Pfulg A Schäublin H Stoffel MH. Effects of housing, perches, genetics, and 25-hydroxycholecalciferol on keel bone deformities in laying hens. Poult Sci (2011) 90(8):1637–44. 10.3382/ps.2011-01379 21753197

[31] Casey-Trott T Heerkens JLT Petrik M Regmi P Schrader L Toscano MJ Methods for assessment of keel bone damage in poultry. Poult Sci (2015) 94(10):2339–50. 10.3382/ps/pev223 26287001

[32] Tarlton JF Wilkins LJ Toscano MJ Avery NC Knott L. Reduced bone breakage and increased bone strength in free range laying hens fed omega-3 polyunsaturated fatty acid supplemented diets. Bone (2013) 52(2):578–86. 10.1016/j.bone.2012.11.003 23142806

[33] Beaupied H Lespessailles E Benhamou C-L. Evaluation of macrostructural bone biomechanics. Joint Bone Spine (2007) 74(3):233–9. 10.1016/j.jbspin.2007.01.019 17382570

[34] Cooper MD Wrathall JHM Assurance schemes as a tool to tackle genetic welfare problems in farm animals. Anim Welf (2010) 19:51–6.

[35] Rath NC Huff GR Huff WE Balog JM. Factors regulating bone maturity and strength in poultry. Poult Sci (2000) 79(7):1024–32. 10.1093/ps/79.7.1024 10901206

[36] Toscano MJ Booth F Wilkins LJ Avery NC Brown SB Richards G The effects of long (C20/22) and short (C18) chain omega-3 fatty acids on keel bone fractures, bone biomechanics, behavior, and egg production in free-range laying hens. Poult Sci (2015) 94(5):823–35. 10.3382/ps/pev048 25771533

[37] Akter M Graham H Iji PA. Response of broiler chickens to different levels of calcium, non-phytate phosphorus and phytase. Br Poult Sci (2016) 57(6):799–809. 10.1080/00071668.2016.1216943 27459412

[38] Regmi P Smith N Nelson N Haut RC Orth MW Karcher DM. Housing conditions alter properties of the tibia and humerus during the laying phase in Lohmann white Leghorn hens. Poult Sci (2016) 95(1):198–206. 10.3382/ps/pev209 26467011

[39] Rodenburg TB Tuyttens FAM de Reu K Herman L Zoons J Sonck B Welfare assessment of laying hens in furnished cages and non-cage systems: an on-farm comparison. Anim Welf (2008) 17(4):363–73.

[40] Gebhardt-Henrich SG Fröhlich EK Early onset of laying and bumblefoot favor keel bone fractures. Animals (Basel) (2015) 5(4):1192–206. 10.3390/ani5040406 26633520 PMC4693210

